# Ganglion Cell – Inner Plexiform Layer Damage in Diabetic Patients: 3-Year Prospective, Longitudinal, Observational Study

**DOI:** 10.1038/s41598-020-58465-x

**Published:** 2020-01-30

**Authors:** Hyung Bin Lim, Yong Il Shin, Min Woo Lee, Hyungmoon Koo, Woo Hyuk Lee, Jung Yeul Kim

**Affiliations:** 1 Department of Ophthalmology, Chungnam National University College of Medicine, Daejeon, Republic of Korea; 2 Department of Ophthalmology, Konyang University Hospital, Daejeon, Republic of Korea

**Keywords:** Diabetes complications, Medical imaging, Retinal diseases

## Abstract

Diabetes is expected to accelerate age-related ganglion cell–inner plexiform layer (GC-IPL) loss, but there is limited information on the rate of reduction in GC-IPL thicknesses. We aimed to evaluate the reduction rate of GC-IPL thickness in diabetic patients, and to compare the rates between patients without and with diabetic retinopathy (DR). We included 112 eyes of 112 patients with diabetes [49 eyes without DR (no-DR group) and 63 eyes with mild to moderate non-proliferative DR (NPDR group)] and 63 eyes of 63 normal controls (control group) in this study. Macular GC-IPL thickness in all participants was measured for 3 years at 1-year intervals. The reduction rates of GC-IPL thickness were determined by linear mixed models and compared among the three groups. The estimated reduction rates of the average GC-IPL thickness in the no-DR (−0.627 μm/year) and NPDR (−0.987 μm/year) groups were 2.26-fold (p = 0.010) and 3.56-fold (p = 0.001) faster, respectively, than the control group (−0.277 μm/year). Age, duration of diabetes, and baseline average GC-IPL thickness were associated with longitudinal changes in average GC-IPL thickness. The GC-IPL reduction rate was significantly faster in diabetic patients, with and without DR. Physicians should therefore be aware that GC-IPL damage continues even if there is no DR.

## Introduction

Diabetic retinopathy (DR), the most common complication of diabetes, is the leading cause of preventable visual impairment^1,2^. In general, DR is clinically defined based on the observation of abnormal fundus vascular lesions, such as microaneurysms, hemorrhages, hard exudates, and cotton wool spots. However, experimental and clinical studies have shown that neurodegenerative changes, including loss of ganglion cells and glial reactivity, are also early events in the pathogenesis of DR^3–5^. The loss of ganglion cells affects retinal ganglion cell layer and retinal nerve fiber layer (RNFL) thickness, and reduced thickness of these layers has been detected in clinical and animal studies^3,6–8^.

Optical coherence tomography (OCT) is a reliable method for quantitative structural evaluation of inner retinal layers, including of the thickness of the peripapillary RNFL (pRNFL) and macular ganglion cell-inner plexiform layer (GC-IPL), where such parameters could be useful for evaluating various conditions, such as retinal neuro-ophthalmic disease and glaucoma^9–11^. Progressive changes in GC-IPL thickness can be identified by serial analysis of OCT measurements, and this trend-based analysis could be useful to understand the pathogenesis of certain conditions.

Although microvascular abnormalities represent the classic hallmarks of DR, recent studies have reported that diabetic retinal neurodegeneration (DRN) occurs in patients without DR and DRN, which antecedes DR^12–15^. The presence of age-related loss of retinal ganglion cells has been demonstrated histologically, and a progressive reduction in GC-IPL thickness, as measured by OCT, has also been reported^16^. Abnormal systemic and eye conditions may accelerate the age-related loss of GC-IPL thickness^17,18^. If acceleration of age-related loss of the GC-IPL occurs in diabetic patients, it may constitute important evidence that neuronal degeneration precedes microvascular changes in diabetic patients.

Reduced GC-IPL thickness in diabetic patients has been reported in several studies. However, most of these studies were cross-sectional, and a basic longitudinal design is also insufficient. We therefore designed this prospective, longitudinal, observational study to investigate the rate of reduction of macular GC-IPL thickness over time in normal controls and diabetic patients, and evaluated the effect of diabetes on progressive macular loss of the GC-IPL.

## Results

### Demographics

A total of 135 diabetic patients and 82 healthy subjects were initially included in this study; 42 individuals were excluded due to follow-up loss (n = 26), intraocular surgery (n = 4), progression of DR (n = 9), or diabetic macular edema (n = 3). As a result, 112 patients (49 in the no-DR group and 63 in the NPDR group) and 63 healthy subjects were finally enrolled.

The mean age of the control, no-DR, and NPDR groups was 56.48 ± 9.30, 59.11 ± 9.35, and 59.05 ± 10.24 years, respectively, and there was no significant difference among the three groups (p = 0.215; Table 1). Sex, hypertension, spherical equivalent (SE), intraocular pressure (IOP), axial length (AL), rim area, cup/disc ratio, and central macular thickness (CMT) were also not significantly different among the groups (all, p > 0.05). The duration of diabetes was 7.1 ± 4.4 and 14.1 ± 8.5 years in the no-DR and NPDR groups, respectively (p < 0.001), and the respective HbA1c levels were 6.9 ± 1.2% and 7.9 ± 1.2%, respectively (p = 0.001). The average GC-IPL thickness in the no-DR group (81.10 ± 4.47 μm; p = 0.024) and NPDR group (80.19 ± 8.99 μm; p = 0.001) was significantly lower than in the control group (84.23 ± 6.22 μm); the respective average pRNFL thickness was 96.23 ± 10.98, 93.49 ± 6.36, and 90.90 ± 8.99 μm (p = 0.003) and, in post hoc analysis, a significant difference was only observed between the control and NPDR groups (p = 0.002).

**Table 1:** Baseline characteristics of the study subjects.

	Control group (n = 63)	No-DR group (n = 49)	NPDR group (n = 63)	p-value (post hoc)
Age (mean ± SD, years)	56.48 ± 9.30	59.11 ± 9.35	59.05 ± 10.24	0.215^*^
Sex (male/female)	24/39	23/26	33/30	0.291^†^
Hypertension (n, %)	18 (28.6%)	17 (34.7%)	23 (36.5%)	0.606
Duration of diabetes (mean ± SD, years)		7.1 ± 4.4	14.1 ± 8.5	**<0.001**^**‡**^
HbA1c (mean ± SD, %)		6.9 ± 1.2	7.9 ± 1.2	**0.001**^**‡**^
BCVA (mean ± SD, logMAR)	−0.03 ± 0.11	−0.02 ± 0.06	0.01 ± 0.06	0.115^*^
Spherical equivalent (mean ± SD, diopters)	1.13 ± 1.44	0.05 ± 1.44	0.96 ± 1.49	0.521^*^
Intraocular pressure (mean ± SD, mmHg)	16.20 ± 2.77	16.59 ± 2.99	16.32 ± 2.85	0.815^*^
Axial length (mean ± SD, mm)	24.58 ± 1.28	24.39 ± 1.43	24.12 ± 1.69	0.451^*^
Rim area (mean ± SD, mm^2^)	1.31 ± 0.19	1.32 ± 0.21	1.39 ± 0.26	0.154^*^
Cup/disc ratio (mean ± SD)	0.54 ± 0.14	0.55 ± 0.11	0.56 ± 0.14	0.192^*^
Central macular thickness (mean ± SD, μm)	253.18 ± 24.66	247.43 ± 21.62	249.52 ± 23.23	0.247^*^
Average GC-IPL thickness (mean ± SD, μm)	84.23 ± 6.22	81.10 ± 4.47	80.19 ± 8.99	0.001^*^ **(Control > no-DR, NPDR)**
Average pRNFL thickness (mean ± SD, μm)	96.23 ± 10.98	93.49 ± 6.36	90.90 ± 8.99	**0.003** **(Control > NPDR)**

All values are the mean ± SD.

SD = standard deviation; HbA1c = hemoglobin A1C; BCVA = best-corrected visual acuity; logMAR = logarithm of the minimum angle of resolution; GC-IPL = ganglion cell-inner plexiform layer; pRNFL = peripapillary retinal nerve fiber layer; DR = diabetic retinopathy; NPDR = nonproliferative diabetic retinopathy.

*p-value for one-way analysis of variance.

^†^p-value for chi-squared test.

^‡^p-value for Student’s *t*-test (no-DR vs. NPDR group).

Significant differences are in bold font.

### The GC-IPL thickness at each visit

**Figure 1:**
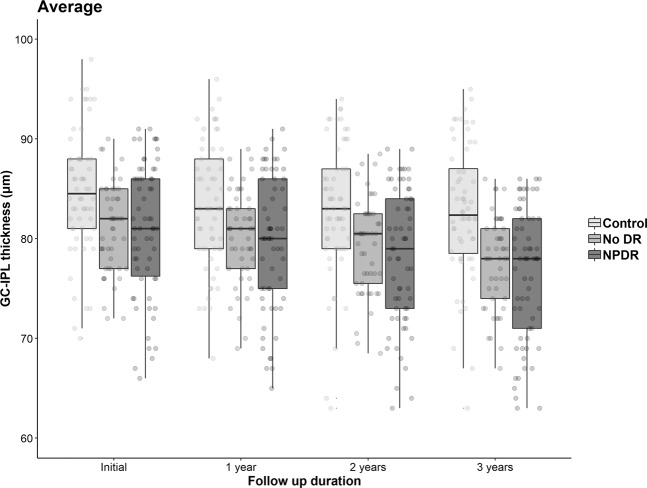
Scatter and box plots of average ganglion cell-inner plexiform layer (GC-IPL) thickness at each clinical visit. Boxes are 25% to 75% (lower to upper) quartiles; the lines in the boxes are medians and the whiskers indicate variability (minimum and maximum values). Average GC-IPL thickness significantly decreased over time in all groups (all, p < 0.05). DR = diabetic retinopathy; NPDR = non-proliferative DR.

The average thickness, and that of the six segments, of the GC-IPL showed a significant reduction during the 3-year period in all three groups (all, p < 0.05), except the superotemporal (p = 0.107), inferotemporal (p = 0.057), and superonasal sector (p = 0.146) thicknesses in the control group. Moreover, significant differences among the three groups were observed at all visits. Using post hoc analysis, the no-DR and NPDR groups had significantly reduced thickness measurements compared with the control group (all, p < 0.05), except at the initial visit with respect to the superior, superotemporal, and inferior sectors, which showed differences only between the control and NPDR groups. No significant difference was found between the no-DR and NPDR groups, at any visit or for any thickness parameter. Details of all these analyses are provided in Table 2 and Fig. 1.

**Table 2:** Changes in ganglion cell-inner plexiform layer thickness by visit.

		Control group (μm)	No-DR group (μm)	NPDR group (μm)	p-value and post-hoc analysis*
Average	Initial	84.23 ± 6.22	81.10 ± 4.47	80.19 ± 8.99	**0.001**^**‡**^
	1-year FU	83.73 ± 6.22	80.27 ± 4.67	79.03 ± 7.65	**<0.001**^**‡**^
	2-year FU	83.56 ± 6.71	79.28 ± 4.70	77.60 ± 8.26	**<0.001**^**‡**^
	3-year FU	83.26 ± 6.76	77.63 ± 4.54	76.48 ± 6.66	**<0.001**^**‡**^
	p-value^†^	**0.012**	**<0.001**	**<0.001**	
Superior segment	Initial	85.27 ± 6.93	82.29 ± 4.88	80.90 ± 9.66	**0.006**^**§**^
	1-year FU	86.22 ± 7.02	81.20 ± 4.93	79.92 ± 8.37	**<0.001**^**‡**^
	2-year FU	84.35 ± 8.17	80.36 ± 4.89	78.48 ± 9.21	**<0.001**^**‡**^
	3-year FU	84.18 ± 7.93	78.57 ± 4.96	77.32 ± 7.69	**<0.001**^**‡**^
	p-value^†^	**<0.001**	**<0.001**	**<0.001**	
Superotemporal segment	Initial	83.47 ± 7.00	80.18 ± 5.38	80.11 ± 8.95	**0.021**^**§**^
	1-year FU	84.23 ± 7.15	79.35 ± 5.41	79.11 ± 8.21	**<0.001**^**‡**^
	2-year FU	82.78 ± 8.11	78.58 ± 5.38	78.03 ± 8.50	**0.001**^**‡**^
	3-year FU	83.05 ± 7.21	76.92 ± 4.92	76.81 ± 6.83	**<0.001**^**‡**^
	p-value^†^	0.107	**<0.001**	**0.001**	
Inferotemporal segment	Initial	84.90 ± 7.20	80.53 ± 5.76	80.84 ± 8.98	**0.003**^**‡**^
	1-year FU	85.50 ± 6.71	79.98 ± 5.68	80.05 ± 6.95	**<0.001**^**‡**^
	2-year FU	83.53 ± 8.12	78.79 ± 6.25	78.95 ± 7.54	**0.002**^**‡**^
	3-year FU	83.83 ± 8.94	77.31 ± 5.64	77.17 ± 6.56	**<0.001**^**‡**^
	p-value^†^	0.057	**<0.001**	**<0.001**	
Inferior segment	Initial	81.52 ± 8.64	78.22 ± 6.12	77.81 ± 9.39	**0.031**^**§**^
	1-year FU	81.98 ± 7.98	77.69 ± 7.05	76.14 ± 9.65	**0.001**^**‡**^
	2-year FU	80.25 ± 10.42	76.15 ± 7.36	75.03 ± 8.88	**0.005**^**‡**^
	3-year FU	80.23 ± 9.64	75.12 ± 5.93	73.84 ± 7.37	**<0.001**^**‡**^
	p-value^†^	**0.030**	**<0.001**	**0.001**	
Inferonasal segment	Initial	85.52 ± 6.12	81.90 ± 4.86	79.94 ± 9.43	**<0.001**^**‡**^
	1-year FU	85.92 ± 7.10	80.94 ± 5.22	78.49 ± 9.46	**<0.001**^**‡**^
	2-year FU	85.17 ± 5.93	79.74 ± 5.00	77.38 ± 9.03	**<0.001**^**‡**^
	3-year FU	84.48 ± 6.71	78.00 ± 5.33	75.92 ± 8.81	**<0.001**^**‡**^
	p-value^†^	**0.014**	**<0.001**	**0.001**	
Superonasal segment	Initial	87.57 ± 6.21	83.73 ± 5.08	82.08 ± 10.21	**<0.001**^**‡**^
	1-year FU	88.08 ± 6.48	82.55 ± 5.30	80.44 ± 9.13	**<0.001**^**‡**^
	2-year FU	87.85 ± 7.79	81.52 ± 5.44	78.44 ± 11.94	**<0.001**^**‡**^
	3-year FU	86.85 ± 6.25	79.78 ± 5.53	77.90 ± 8.85	**<0.001**^**‡**^
	p-value^†^	0.146	**<0.001**	**<0.001**	

All values are the mean ± standard deviation.

DR = diabetic retinopathy; NPDR = nonproliferative diabetic retinopathy; FU, follow-up.

*p-value for one-way analysis of variance (ANOVA) with Bonferroni correction.

^†^p-value for repeated-measures ANOVA.

^**‡**^Indicates control group was significantly greater than the no-DR and NPDR groups.

^**§**^Indicates control group was significantly greater than the NPDR group.

Significant differences are in bold font.

### Rate of reduction of GC-IPL thickness

The estimated rate of reduction of the average GC-IPL thickness, in the control, no-DR, and NPDR groups, was −0.277 (95% CI: −0.445, −0.108; p = 0.001), −0.627 (95% CI: −0.779, −0.475; p < 0.0001), and −0.987 μm/year (95% CI: −1.336, −0.637; p < 0.0001; Table 3), respectively, and the rate in two diabetic groups were 2.26-fold (p = 0.010) and 3.56-fold (p = 0.001) faster than the control group. The estimated mean reduction rate was significant for all parameters, in all three groups (all, p < 0.05), except for the superotemporal (p = 0.834), inferotemporal (p = 0.070), and superonasal (p = 0.097) sector thicknesses in the control group. When comparing the rate of reduction of the thickness parameters among the three groups, the no-DR group showed a higher reduction rate in the average (p = 0.010), superotemporal (p = 0.001), inferonasal (p = 0.032), and superonasal (p = 0.042) values than the control group. The NPDR group showed a higher reduction rate than the control group in all thickness parameters (all, p < 0.05), whereas no difference was found in the no-DR group versus the NPDR group (all, p > 0.05).

**Table 3:** Estimated mean rates of reduction in the ganglion cell-inner plexiform layer thickness: results from linear mixed models.

	Control (μm/year, 95% CI)	p-value	No-DR (μm/year, 95% CI)	p-value	NPDR (μm/year, 95% CI)	p-value	p-value * (control vs. no-DR)	p-value * (control vs. NPDR)	p-value * (no-DR vs. NPDR)
Average	−0.277 (−0.445, −0.108)	**0.001**	−0.627 (−0.779, −0.475)	**<0.0001**	−0.987 (−1.336, −0.637)	**<0.0001**	**0.010**	**0.001**	0.163
Superior	−0.301 (−0.577, −0.025)	**0.033**	−0.614 (−0.894, −0.335)	**<0.0001**	−0.929 (−1.266, −0.592)	**<0.0001**	0.126	**0.004**	0.230
Superotemporal	−0.026 (−0.276, 0.223)	0.834	−0.588 (−0.771, −0.405)	**<0.0001**	−0.806 (−1.181, −0.432)	**<0.0001**	**0.001**	**0.0005**	0.452
Inferotemporal	−0.308 (−0.641, 0.025)	0.070	−0.593 (−0.769, −0.417)	**<0.0001**	−0.899 (−1.238, −0.561)	**<0.0001**	0.196	**0.009**	0.224
Inferior	−0.416 (−0.824, −0.009)	**0.045**	−0.573 (−0.834, −0.312)	**<0.0001**	−1.039 (−1.479, −0.599)	**<0.0001**	0.614	**0.034**	0.151
Inferonasal	−0.352 (−0.605, −0.099)	**0.007**	−0.719 (−0.889, −0.550)	**<0.0001**	−1.047 (−1.484, −0.610)	**<0.0001**	**0.032**	**0.007**	0.300
Superonasal	−0.269 (−0.587, 0.049)	0.097	−0.700 (−0.902, −0.498)	**<0.0001**	−1.161 (−1.614, −0.709)	**<0.0001**	**0.042**	**0.001**	0.163

DR = diabetic retinopathy; NPDR = nonproliferative DR; CI = confidence interval.

*p-value for between-group differences and the follow-up duration based on linear mixed models.

Significant differences are in bold font.

### Factors associated with longitudinal changes of GC-IPL thickness

In univariate linear mixed models, age (p = 0.001), duration of diabetes (p = 0.005), BCVA (p < 0.001), and baseline average GC-IPL thickness (p < 0.001) were significantly associated with longitudinal changes in the average GC-IPL thickness (Table 4). In multivariate linear mixed models including the above significant variables in univariate analyses, age (p = 0.019), duration of diabetes (p = 0.016), and baseline average GC-IPL thickness (p < 0.001) were again significantly associated with longitudinal changes in the average GC-IPL thickness.

**Table 4:** Univariate and multivariate linear mixed model of factors associated with changes in average ganglion cell-inner plexiform layer thickness over time.

	Univariate	p-value	Multivariate	p-value
	Estimate (μm/year, 95% CI)		Estimate (μm/year, 95% CI)	
Age (years)	−0.155 (−0.247, −0.064)	**0.001**	−0.041 (−0.073, −0.007)	**0.019**
Female sex	0.799 (−1.199, 2.798)	0.431		
Duration of diabetes (years)	−0.628 (−0.829, −0.420)	**0.005**	−0.712 (−1.026, −0.254)	**0.016**
HbA1c (%)	−0.618 (−1.423, 0.187)	0.131		
BCVA (logMAR)	−20.454 (−30.986, −9.921)	**<0.001**	−2.550 (−6.541, 1.442)	0.209
Intraocular pressure (mmHg)	0.156 (−0.203, 0.514)	0.367		
Spherical equivalent (diopter)	−0.191 (−0.665, 0.284)	0.429		
Axial length (mm)	−0.682 (−2.065, 0.701)	0.328		
Baseline average GC-IPL (μm)	0.854 (0.804, 0.904)	**<0.001**	0.830 (0.780, 0.881)	**<0.001**

HbA1c = hemoglobin A1C; BCVA = best-corrected visual acuity; logMAR = logarithm of the minimum angle of resolution; GC-IPL = ganglion cell-inner plexiform layer; CI = confidential interval.

Significant differences are in bold font.

## Discussion

In this prospective study, we followed-up 112 diabetic patients and 63 normal controls for 3 years and analyzed longitudinal changes in macular GC-IPL thickness. The results showed a significant reduction in GC-IPL thickness over time, and the rate of reduction of the average GC-IPL thickness in the no-DR and NPDR groups was 2.26- and 3.56-fold faster than that in the normal control group, respectively; this confirms that diabetes is a critical factor accelerating age-related loss of the GC-IPL thickness, where this was observed regardless of whether DR was present.

Retinal blood flow is affected by neural activity and retinal metabolism, which is known as neurovascular coupling and involves complex mechanisms^19^. In diabetic patients, hyperglycemia triggers metabolic pathways, such as the polyol and hexosamine pathways, resulting in the production of free radicals and advanced glycation end products; along with inflammation and ischemia, these are essential processes for the development of DR^20,21^. The activation of these pathways causes abnormalities in the neural retina, resulting in retinal neurodegeneration and retinal microangiopathy in the capillary bed. The most important features of DRN are neural apoptosis and reactive gliosis^3^. A previous study reported that retinal ganglion and amacrine cells were the retinal neurons associated with apoptosis, the process of which is accompanied by reactive gliosis occurring in astrocytes and Müller cells^22,23^. These processes cause a reduction in the thickness of the inner retinal layer, including the GC-IPL and RNFL, which can be detected by OCT.

Recent studies have suggested that DRN occurs prior to vascular abnormalities in diabetic patients, and is involved in the development of early microvascular changes. Neurodegeneration has been shown to cause breakdown of the blood-retinal barrier (BRB)^24,25^, as well as vasoregression^26^ and impairment of neurovascular coupling^27,28^. In addition, glutamate accumulation induced by DRN increases the secretion of vascular endothelial growth factor, which leads to damage to the BRB^29^. Considering these mechanisms, DRN is a crucial factor in the development of DR, and it could explain our finding of significant GC-IPL loss in patients without DR.

Although ganglion cell loss in diabetic patients has been identified by histological and experimental studies^4^, cross-sectional studies using OCT have reported different results. Studies by van Dijk *et al*.^5,8,30^ reported a decrease in GC-IPL and macular RNFL thickness in patients with, but not without, DR. However, Ng *et al*.^31^ reported GC-IPL loss in patients both with and without DR; the loss was progressive in advanced DR, with a decrease in inner retinal layer thickness seen in both the no-DR and NPDR groups. The retinal ganglion cell layer has been associated with DRN and may show changes that can be detected, because the ganglion cell layer in the macular area is 10–20 times thicker than their axons in the RNFL. In addition, several abnormalities in retinal function have been observed in diabetic patients and rats without DR, based on electroretinograms^32–34^. Considering the above results and those of the present study, which showed a significantly higher rate of reduction of GC-IPL thickness in the no-DR group than in the control group, inner retinal thinning in patients without DR supports the view that DRN is an early event in the pathogenesis of DR that precedes retinal microvascular changes.

Although the rate of reduction of GC-IPL thickness in this study was higher in the NPDR group than in the no-DR group, there was no significant difference between the groups, nor in any other thickness parameter. This could be explained by insufficient statistical power to reveal differences between the groups, due to the relatively small group sizes. In addition, GC-IPL loss may have been masked by subclinical macular edema not detected by OCT.

**Figure 2:**
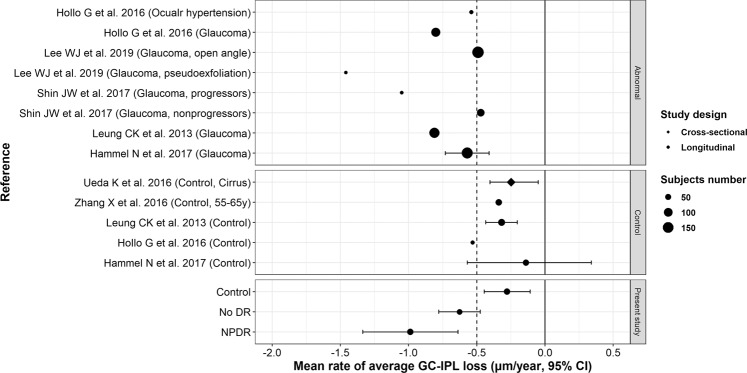
Forest plot of published data pertaining to the average rate of GC-IPL loss in abnormal and normal eyes. The mean rate of change of GC-IPL thickness and its 95% confidential interval are denoted as points and lines, respectively. Point shape and size denote study design and number of subjects, respectively.

The results of this study were similar to our previous study, which studied pRNFL thickness using a similar study design^35^. The previous study determined that diabetic eyes had a significantly greater decrease in pRNFL over 3 years than normal eyes regardless of mild DR progression. Several previous studies have reported progressive age-related loss of GC-IPL thickness, based on spectral domain OCT, in normal subjects and patients with certain ocular conditions (Fig. 2). The average rate of GC-IPL thickness reduction in normal subjects was reported as −0.249 ~ −0.53 μm/year^36–39^, whereas Hanmmel *et al*.^40^ reported no significant age-related reduction. These values were then compared with those for abnormal conditions, particularly glaucoma, and the rate of reduction in glaucoma patients was found to be significantly higher than that in normal subjects. There was also a difference in the rate according to glaucoma status, which was reported to be in the range of approximately −0.5 ~ −1.0 μm/year^38–41^, and Lee *et al*.^42^ reported a rate of −1.46 μm/year in pseudoexfoliation glaucoma. The rate of GC-IPL loss in the control group in the present study (−0.277 µm/year) was consistent with the above studies, and the reduction rates in the no-DR and NPDR groups (−0.627 µm/year and −0.987 µm/year, respectively) were similar to those of glaucoma patients. Although it is difficult to directly compare these values because of differences in study design, our results suggested that diabetes is a significant factor in GC-IPL loss, with an importance comparable to that of glaucoma.

This study had several limitations. First, the longitudinal data on blood glucose levels were not obtained. Although the initial HbA1c levels were not associated with changes in GC-IPL thickness, and no relationship between serial HbA1c levels and progression of DRN was reported in the previous study^43^, longitudinal changes in HbA1c levels might have affected the rate of GC-IPL reduction. Second, inner and outer nuclear layers also expected to change considering the mechanism of DR. However, we did not analyze because nuclear layer thicknesses cannot be measured automatically on our platform. Additional research will be needed for to investigate the retinal nuclear layer. Third, although we carefully checked for glaucomatous findings, such as RNFL defects and glaucomatous optic disc based on OCT findings, we did not perform a visual field test and the patient’s data was not reviewed by a glaucoma specialist; thus, it is possible that we enrolled patients with pre-perimetric glaucoma. Finally, we could not analyze the association between DRN and functional changes. Additional well-designed prospective studies are needed to overcome these limitations.

In conclusion, we confirmed that the GC-IPL reduction rate was significantly faster in patients with diabetes, both without and with DR, compared with normal controls. This is the first study to demonstrate a temporal association of GC-IPL reduction with progression of diabetes. Our results suggest that DRN antecedes the microvasculopathy in people with DR, in turn suggesting that DRN may be an important component in the pathogenesis of the GC-IPL reductions. Our results improve understanding of the pathophysiology of GC-IPL changes in diabetic patients, and should be valuable in the analysis of GC-IPL thickness in patients with glaucoma or neuroretinal disease. Physicians should therefore consider the effects of diabetes on the GC-IPL.

## Methods

### Subjects

This investigation was a prospective, longitudinal, observational study involving patients with diabetes. The study was approved by the Chungnam National University Hospital Institutional Review Board (Daejeon, Republic of Korea), and conformed to the tenets of the Declaration of Helsinki. Written informed consent was obtained from all subjects prior to participation. Methodological details of our study have been described previously^35^.

Patients with diabetes were enrolled consecutively at the Retina and Vitreous Clinic of Chungnam National University Hospital between January 2013 and June 2015. All patients were diagnosed with diabetes initially at the Department of Internal Medicine of Chungnam National University Hospital according to the criteria of the American diabetes association^44^. Eligible participants had a best-corrected visual acuity (BCVA) of 20/25 or better in the study eye. The exclusion criteria included patients with a history of systemic disease other than hypertension and diabetic mellitus, history or evidence of ocular surgery, glaucoma, IOP > 21 mmHg, optic nerve disorders, AL ≥ 26.0 mm, SE >  + 6.0 diopters (D) or < −6.0 D, optic disc abnormalities, or any other retinal dysfunction. If both eyes met the eligible criteria, one eye was selected randomly.

All participants initially underwent a comprehensive ophthalmic examination, including a review of the patient’s medical history, BCVA, IOP measurement, slit-lamp examination, dilated fundus examination, AL using the IOLMaster^®^ (Carl Zeiss Meditec, Jena, Germany), photography, OCT (Carl Zeiss Meditec, Dublin, CA, USA), and fluorescein angiography using an HRA Spectralis system (Heidelberg Engineering, Heidelberg, Germany). We divided the diabetic patients into two groups: a no-DR group and a nonproliferative DR (NPDR) group. NPDR was graded according to the International Clinical Diabetic Retinopathy Disease Severity scale^45^. The NPDR group consisted only of mild to moderate NPDR cases, and all patients with a more advanced grade of DR, such as severe NPDR or proliferative DR were excluded.

Among the subjects who visited our Retina and Vitreous clinic for various reasons (routine checkups for ocular diseases such as peripheral vitreous floaters, cataract, health screening checkups), those who met eligibility criteria and had glucose level data [fasting plasma glucose < 100 mg/dL or hemoglobin A1C (HbA1c) < 5.7%] were enrolled in the normal control group. Normal subjects (control group) had no history of diabetes and no ocular disease, with a BCVA ≥ 20/25, a normal IOP range, and a SE within ± 6.0 D.

All participants underwent examinations including BCVA, IOP, slit-lamp examination, dilated fundus examination, photography, and OCT every 12 months for 36 months (total of four examinations). Patients who were diagnosed with progressed DR grade (except mild to moderate NPDR) or macular edema, were lost to follow up, or underwent intraocular surgery were excluded.

### OCT protocol

**Figure 3:**
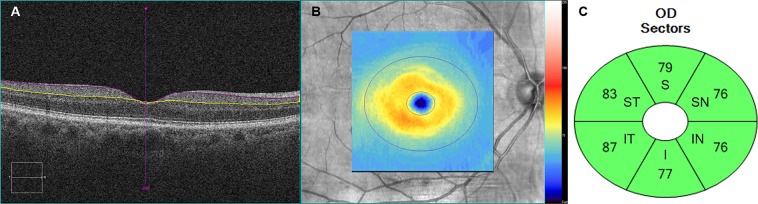
Cirrus optical coherence tomography images of macula of the right eye: (**A**) horizontal scan of the macular showing a segmented ganglion cell – inner plexiform layer (measured between the purple and yellow horizontal lines), (**B**) color-coded topographic map, (**C**) six sectors [superior (S), superotemporal (ST), inferotemporal (IT), inferior (I), inferonasal (IN), superonasal (SN)] of the elliptical annulus.

Spectral domain OCT scan was performed using a Cirrus HD-OCT^®^ instrument (Carl Zeiss). In the macular scan, GC-IPL thickness was measured using the ganglion cell analysis algorithm in the macular cube 200 × 200 protocol^46^. This algorithm calculates the average thickness of six sectors (superotemporal, superior, superonasal, inferonasal, inferior, and inferotemporal) of the elliptical annulus (vertical inner and outer radii of 0.5 and 2.0 mm, respectively; horizontal inner and outer radii of 0.6 and 2.4 mm, respectively), centered on the macular area (Fig. 3). The pRNFL thickness and optic nerve head (ONH) parameters, such as the rim area and average cup/disc ratio were measured using the optic disc 200 × 200 protocol. All participants underwent two OCT scans by an experienced examiner in all participants, and we selected the best scan with signal strength ≥ 7. We excluded results from patients with OCT scan signal strength < 7 or segmentation errors.

### Statistical analyses

All statistical analyses were performed using the statistical package R (version 3.5.0; R Foundation for Statistical Computing, Vienna, Austria) and SPSS statistical software (version 21.0; SPSS Inc., Chicago, IL, USA). Snellen BCVA results were converted into the logarithm of the minimum angle of resolution (logMAR). Continuous variables are presented as the mean ± standard deviation. Differences were considered significant at p < 0.05.

Baseline demographics and OCT measurements, including CMT, average GC-IPL, average pRNFL thickness, and ONH parameters were compared among groups using one-way analysis of variance (ANOVA) with post-hoc Bonferroni correction and the chi-square test; GC-IPL thickness at each visit was also compared. Repeated measures ANOVA was used to analyze longitudinal changes in GC-IPL thickness in each group. Linear mixed effects models were used to calculate the rate of reduction, with 95% confidence interval (CI), of GC-IPL thickness over time; the rate was then compared among the three groups. Age, sex, SE, AL, follow-up duration, and interaction among the groups were included as fixed effects, and the subject was included as a random effect. In addition, univariate and multivariate linear mixed models were also fitted to determine factors correlated with longitudinal changes in GC-IPL thickness.

## Data Availability

Data supporting the findings of the current study are available from the corresponding author on reasonable request.

## References

[1] Cheung N, Mitchell P, Wong TY (2010). Diabetic retinopathy. Lancet.

[2] Fong DS (2004). Retinopathy in diabetes. Diabetes care.

[3] Simo R, Hernandez C (2014). Neurodegeneration in the diabetic eye: new insights and therapeutic perspectives. Trends Endocrinol. Metab..

[4] Kern TS, Barber AJ (2008). Retinal ganglion cells in diabetes. J. Physiol..

[5] van Dijk HW (2012). Early neurodegeneration in the retina of type 2 diabetic patients. Invest. Ophthalmol. Vis. Sci..

[6] Cui RZ (2019). ON-Type Retinal Ganglion Cells are Preferentially Affected in STZ-Induced Diabetic Mice. Invest. Ophthalmol. Vis. Sci..

[7] Ha Y (2012). Diabetes accelerates retinal ganglion cell dysfunction in mice lacking sigma receptor 1. Mol. Vis..

[8] van Dijk HW (2010). Decreased retinal ganglion cell layer thickness in patients with type 1 diabetes. Invest. Ophthalmol. Vis. Sci..

[9] Danesh-Meyer HV, Yap J, Frampton C, Savino PJ (2014). Differentiation of compressive from glaucomatous optic neuropathy with spectral-domain optical coherence tomography. Ophthalmol..

[10] Mwanza JC (2012). Glaucoma diagnostic accuracy of ganglion cell-inner plexiform layer thickness: comparison with nerve fiber layer and optic nerve head. Ophthalmol..

[11] Lim HB, Sung JY, Ahn SI, Jo YJ, Kim JY (2018). Retinal Nerve Fiber Layer Thickness in Various Retinal Diseases. Optom. Vis. Sci..

[12] Antonetti DA (2006). Diabetic retinopathy: seeing beyond glucose-induced microvascular disease. Diabetes.

[13] Simo R, Hernandez C (2012). Neurodegeneration is an early event in diabetic retinopathy: therapeutic implications. Br. J. Ophthalmol..

[14] Barber AJ (2003). A new view of diabetic retinopathy: a neurodegenerative disease of the eye. Prog. Neuropsychopharmacol. Biol. Psychiatry.

[15] Lieth E, Gardner TW, Barber AJ, Antonetti DA (2000). Retinal neurodegeneration: early pathology in diabetes. Clin. Exp. Ophthalmol..

[16] Leung CK (2012). Retinal nerve fiber layer imaging with spectral-domain optical coherence tomography: a prospective analysis of age-related loss. Ophthalmol..

[17] Lee MW, Kim JM, Shin YI, Jo YJ, Kim JY (2019). Longitudinal Changes in Peripapillary Retinal Nerve Fiber Layer Thickness in High Myopia: A Prospective, Observational Study. Ophthalmol..

[18] Lee WJ, Kim YK, Park KH, Jeoung JW (2017). Trend-based Analysis of Ganglion Cell-Inner Plexiform Layer Thickness Changes on Optical Coherence Tomography in Glaucoma Progression. Ophthalmol..

[19] Antonetti DA, Klein R, Gardner TW (2012). Diabetic retinopathy. N. Engl. J. Med..

[20] Tang J, Kern TS (2011). Inflammation in diabetic retinopathy. Prog. Retin. Eye Res..

[21] Brownlee M (2001). Biochemistry and molecular cell biology of diabetic complications. Nat..

[22] Carrasco E (2007). Lower somatostatin expression is an early event in diabetic retinopathy and is associated with retinal neurodegeneration. Diabetes Care.

[23] Simo R, Stitt AW, Gardner TW (2018). Neurodegeneration in diabetic retinopathy: does it really matter?. Diabetologia.

[24] Kusari J, Zhou S, Padillo E, Clarke KG, Gil DW (2007). Effect of memantine on neuroretinal function and retinal vascular changes of streptozotocin-induced diabetic rats. Invest. Ophthalmol. Vis. Sci..

[25] Silva KC, Rosales MA, Biswas SK, Lopes de Faria JB (2009). & Lopes de Faria, J. M. Diabetic retinal neurodegeneration is associated with mitochondrial oxidative stress and is improved by an angiotensin receptor blocker in a model combining hypertension and diabetes. Diabetes.

[26] Feng Y (2009). Vasoregression linked to neuronal damage in the rat with defect of polycystin-2. PLoS One.

[27] Luu CD, Szental JA, Lee SY, Lavanya R, Wong TY (2010). Correlation between retinal oscillatory potentials and retinal vascular caliber in type 2 diabetes. Invest. Ophthalmol. Vis. Sci..

[28] Lecleire-Collet A (2011). Evaluation of retinal function and flicker light-induced retinal vascular response in normotensive patients with diabetes without retinopathy. Invest. Ophthalmol. Vis. Sci..

[29] Murata T (1996). The relation between expression of vascular endothelial growth factor and breakdown of the blood-retinal barrier in diabetic rat retinas. Lab. Invest..

[30] van Dijk HW (2009). Selective loss of inner retinal layer thickness in type 1 diabetic patients with minimal diabetic retinopathy. Invest. Ophthalmol. Vis. Sci..

[31] Ng DS (2016). Retinal ganglion cell neuronal damage in diabetes and diabetic retinopathy. Clin. Exp. Ophthalmol..

[32] Ewing FM, Deary IJ, Strachan MW, Frier BM (1998). Seeing beyond retinopathy in diabetes: electrophysiological and psychophysical abnormalities and alterations in vision. Endocr. Rev..

[33] Shirao Y, Kawasaki K (1998). Electrical responses from diabetic retina. Prog. Retin. Eye Res..

[34] Di Leo MA (1990). Spatial frequency-selective losses with pattern electroretinogram in type 1 (insulin-dependent) diabetic patients without retinopathy. Diabetologia.

[35] Lim HB, Shin YI, Lee MW, Park GS, Kim JY (2019). Longitudinal Changes in the Peripapillary Retinal Nerve Fiber Layer Thickness of Patients With Type 2 Diabetes. JAMA Ophthalmol..

[36] Ueda K (2016). Effects of Axial Length and Age on Circumpapillary Retinal Nerve Fiber Layer and Inner Macular Parameters Measured by 3 Types of SD-OCT Instruments. J. Glaucoma.

[37] Zhang X (2016). Longitudinal and Cross-Sectional Analyses of Age Effects on Retinal Nerve Fiber Layer and Ganglion Cell Complex Thickness by Fourier-Domain OCT. Transl. Vis. Sci. Technol..

[38] Leung CKS (2013). Impact of age-related change of retinal nerve fiber layer and macular thicknesses on evaluation of glaucoma progression. Ophthalmol..

[39] Hollo G, Zhou Q (2016). Evaluation of Retinal Nerve Fiber Layer Thickness and Ganglion Cell Complex Progression Rates in Healthy, Ocular Hypertensive, and Glaucoma Eyes With the Avanti RTVue-XR Optical Coherence Tomograph Based on 5-Year Follow-up. J. Glaucoma.

[40] Hammel N (2017). Comparing the Rates of Retinal Nerve Fiber Layer and Ganglion Cell-Inner Plexiform Layer Loss in Healthy Eyes and in Glaucoma Eyes. Am. J. Ophthalmol..

[41] Shin JW, Sung KR, Lee GC, Durbin MK, Cheng D (2017). Ganglion Cell-Inner Plexiform Layer Change Detected by Optical Coherence Tomography Indicates Progression in Advanced Glaucoma. Ophthalmol..

[42] Lee WJ, Baek SU, Kim YK, Park KH, Jeoung JW (2019). Rates of Ganglion Cell-Inner Plexiform Layer Thinning in Normal, Open-Angle Glaucoma and Pseudoexfoliation Glaucoma Eyes: A Trend-Based Analysis. Invest. Ophthalmol. Vis. Sci..

[43] Sohn EH (2016). Retinal neurodegeneration may precede microvascular changes characteristic of diabetic retinopathy in diabetes mellitus. Proc. Natl Acad. Sci. USA.

[44] 2. Classification and Diagnosis of Diabetes: Standards of Medical Care in Diabetes-2018. *Diabetes Care* **41**, S13-s27, 10.2337/dc18-S002 (2018). 10.2337/dc18-S002 29222373

[45] Wilkinson CP (2003). Proposed international clinical diabetic retinopathy and diabetic macular edema disease severity scales. Ophthalmol..

[46] Mwanza JC (2011). Profile and predictors of normal ganglion cell-inner plexiform layer thickness measured with frequency-domain optical coherence tomography. Invest. Ophthalmol. Vis. Sci..

